# Induction of Interleukin-8 from Nasal Epithelial Cells during Bacterial Infection: The Role of IL-8 for Neutrophil Recruitment in Chronic Rhinosinusitis

**DOI:** 10.1155/2010/813610

**Published:** 2010-06-20

**Authors:** Bit-Na Yoon, Nan-Geum Choi, Hyun-Sun Lee, Kyu-Sup Cho, Hwan-Jung Roh

**Affiliations:** Department of ORL-HNS and Medical Research Institute, Pusan National University School of Medicine, Beomeo-ri, Mulgeum-eup, Yangsan-si, Gyeongsangnam-do 626-770, Republic of Korea

## Abstract

*Objectives*. The aim of this study was to elucidate the role of IL-8 for neutrophil recruitment in nonallergic CRS patients. *Methods*. After coculture of *Streptococcus pneumoniae* (SP) with the mucosal epithelial cells (MECs) from non-CRS patients, at three different SP/MEC (1/1, 10/1, 100/1) ratios, the expression of IL-8 mRNA and the concentration of IL-8 were measured by RT-PCR and ELISA. The expression of CD11b/CD18 on neutrophils and E-selectin/ICAM-1 on endothelial cells and the adherence between neutrophils and human umbilical vascular endothelial cells (HUVECs) were determined by flow cytometric analysis, ELISA, and RIA, respectively. *Results*. IL-8 concentration and IL-8 mRNA expression continued to increase from 3 hours after incubation in SP number-dependent manner. The expression of CD11b/CD18 on neutrophils and E-selectin/ICAM-1 on HUVECs, and the adherence between neutrophils and HUVECs were significantly increased in 10 SP/MEC-CM, and the increments were significantly blocked by anti-IL-8 antibody. *Conclusion*. MEC and IL-8 are major factors for neutrophil recruitment in nonallergic CRS.

## 1. Introduction

Histopathologically, nonallergic chronic rhinosinusitis (CRS) is characterized by neutrophil infiltration into sinus mucosa and most of the cells in the sinus effusion and nasal discharge are neutrophils [1]. The neutrophils are known as not only terminal effector cells for tissue destruction and antibacterial defense but also as immunocompetent cells that play an active role in the up-regulation of the inflammatory response by secreting a variety of cytokines such as IL-1*α*, IL-1*β*, IL-6, interferon-*α*, TNF, and IL-8 [2].

Interleukin-8 (IL-8) is an inflammatory cytokine that participates in the pathogenesis of several neutrophil-infiltrating chronic inflammatory diseases such as bronchiectasis, diffuse panbronchiolitis, and cystic fibrosis [3]. It can be secreted by any cell with toll-like receptors such as nasal epithelial cells, nasal gland duct cells, and recruited neutrophils [4]. Previous studies have shown that IL-8 and neutrophils are highly detected in the nasal discharge and sinus mucosa of CRS patients, and they have positive feedback interactions with each other [3, 5].

The recruitment of neutrophils from the circulation into the extravascular space involves several steps. Recent advances in sinusitis research have revealed two positive feedback mechanisms that explain chronic neutrophil accumulation in the sinus effusion [3]. One is the transendothelial migration of neutrophils to the inflammatory sinus mucosa. The other is the emigration of neutrophils out of the mucosa into the sinus effusion. Even though IL-8 has been known as primarily as a potent chemotactic factor for neutrophils, its role for transendothelial migration of neutrophils in CRS has not yet been clearly understood.

To elucidate the role of IL-8 for neutrophil recruitment in nonallergic CRS, we studied the production and biological activity of IL-8 from sinus mucosal epithelial cells (MECs) and IL-8 mediated neutrophil-endothelial interaction after bacterial infection.

## 2. Materials and Methods

### 2.1. Patients

The study group consisted of 20 patients with CRS (12 males and 8 females, age range from 17 to 59) that underwent endoscopic sinus surgery. CRS was defined based on the guidelines established by The Sinus and Allergy Health Partnership. The diagnosis was supported by the clinical history, endoscopic examination, and PNS CT. All of the subjects had normal blood IgE levels and negative results on the multiple antigen simultaneous test (MAST). Patients who had taken antibiotics or corticosteroids during the 4 weeks before surgery were excluded. This study was approved by the Institutional Review Board of Pusan National University (IRB#2007108).

### 2.2. Primary Culture of MECs

Ethmoidal and maxillary sinus mucosa was obtained during the endoscopic sinus surgery. The obtained mucosa was treated with 0.1% pronase (type XIV protease, Sigma Chemical Co., St. Louis, MO) in a culture medium at 4°C for 16–24 hours for dissociation of the MECs. After washing the dissociated cells with culture medium, the cells were plated on a 100 mm Petri dish with culture medium at 37°C for 1 hour to remove fibroblasts, myocytes, and endothelial cells. Then the harvested MECs in the supernatant were grown with culture medium in a 5% CO_2_ incubator at 37°C. The components of the culture medium were a 1 : 1 mixture of Dulbecco's modified Eagle medium and Ham's nutrient F12 (DMEM/F12) including penicillin (100 U/ml), streptomycin (50 *μ*g/ml), cholera toxin (10 ng/ml), retinoic acid (0.1 *μ*M), and 10% NU serum (Collaborative Research Inc., Bedford, MA).

The MECs were washed with Hank's balanced salt solution (HBSS) after detachment with 0.25% trypsin-0.02% EDTA in the confluent state. Thereafter, the MECs were seeded in 6-well tissue culture plates at 3 × 10^4^ cells per well in a volume of 3 ml per well and cultured for 4 days.

### 2.3. Bacterial Strain and Culture Conditions

Streptococcus pneumoniae (SP), used to induce IL-8 production in this study, was isolated from a patient with upper respiratory tract illness. The organism was inoculated on a blood agar plate and was grown in a 5% CO_2_ incubator, at 37°C. After 72 hours, bacterial cells were harvested with sterile cotton swabs into saline, using 1 ml per plate (approximately 10^9^ − 10^10^ bacteria). Next, they were washed with saline. The bacteria were resuspended in antibiotics-free tissue culture medium (it was the same as epithelial cell culture medium except for the absence of antibiotics), and added to the cultured MECs at various SP/MEC (1/1–100/1) ratios in a 1-ml volume.

### 2.4. Induction of IL-8

To measure IL-8 production, from the MECs after SP infection, first the MEC monolayer was infected with SP by adding SP in three different SP/MEC (1/1, 10/1, 100/1) ratios in a 6-well tissue culture plate. The tissue culture plates were maintained at 37°C for 24 hours. The level of cytokines (IL-6, IL-8, TNF-*α*) and expression of IL-8 mRNA were assayed by an enzyme-linked immunosorbent assay (ELISA) and reverse transcription-polymerase chain reaction (RT-PCR) in the conditioned medium of MEC (MEC-CM) and MEC co-cultured with SP (SP/MEC-CM) at three different SP/MEC ratios.

### 2.5. RT-PCR

The total cellular RNA was extracted from the MECs using an RNA-zol kit according to the manufacturer's instructions (Biotec Laboratories Inc., Friendswood, TX). The RNA (2 *μ*g) was reverse transcripted, and the complementary DNA was amplified in a 50 ul reaction by Perkin Elmer thermocycler for 30 cycles with 15 ul of commercially available PCR primers for IL-8 (Clontech Laboratories, Inc., Palo Alto, CA). The sequence for the 5′ primer was ATG ACT TCC AAG CTG GCC GTG GCT (+1 to +24) and the 3′ primer was T CTC AGC CCT CTT CAA AAA CTT CTC (+289 to +264). Negative and positive controls were included in each experiment. The PCR product was separated by electrophoresis on a 1.5% agarose gel.

### 2.6. Cytokine ELISA Assays

IL-6, IL-8, and TNF-*α* were quantitated using a commercial ELISA kit according to the manufacturer's instructions (R&D Systems, Minneapolis, MN). The detection limits of the ELISA for IL-6, IL-8, and TNF-*α* were 35, 80, and 70 pg/ml, respectively.

### 2.7. Isolation and ^51^Cr-Labeling of Neutrophils

Human neutrophilic polymorphonuclear leukocytes were isolated from the venous blood of healthy adults using standard dextran sedimentation and gradient separation with Histopaque 1077 (Sigma Diagnostic, Inc. St. Louis, Mo). The isolated neutrophils were suspended in PBS (2 × 10^7^ cells/ml) and radiolabeled by incubating the cells, with 30 uCi Na^51^CrO_4_/ml at 37°C, for 60 min. The cells were washed twice with cold PBS, at 250 g for 8 min, to remove unincorporated radioactivity and then resuspended in plasma-free HBSS.

### 2.8. Preparation of Endothelial Cells

Human umbilical vascular endothelial cells (HUVECs, CRL-1730, ATCC) were plated in Medium 199 (Gibco Lab, Grand Island, NY) supplemented with 10% heat-inactivated fetal calf serum, heparin sodium (90 *μ*g/ml), penicillin (100 IU/ml), streptomycin (100 *μ*g/ml), and endothelial cell growth supplement (100 *μ*g/ml). The cells were seeded into 0.1% gelatin-coated tissue culture plate and incubated with 5% CO_2_ at 37°C in 95% humidity and were used when confluent.

### 2.9. Measurement of the Expression of CD11b/CD18 (Mac-1) on Neutrophils

Purified neutrophils (1 × 10^6^) were incubated with 0.1 ml of MEC-CM and 10 SP/MEC-CM for 15 min at 37°C. Subsequently, the cells were washed and stained with FITC-labeled antihuman CD11b/CD18 (Anti-Leu-15, Becton Dickinson, San Jose, CA), for 30 min at 4°C. They were then examined by flow cytometry (FACSort, Becton Dickinson, San Jose, CA). To confirm the specificity of the changes induced by IL-8, in the SP/MEC-CM, 100 *μ*g/ml of goat antihuman IL-8 IgG (Becton Dickinson, San Jose, CA) or identical amounts of goat IgG for isotype control (Becton Dickinson) were added to the 10 SP/MEC-CM before incubation with the neutrophils. The data were expressed as a percentage of fluorescence based on the intensity of the surface antigen expression compared to 100% fluorescence in the control (neutrophils in MEC-CM).

### 2.10. Measurement of the Expression of the Endothelial Adhesion Molecules

Cell surface expressions of E-selectin and ICAM-1 were determined using a whole cell ELISA. HUVECs were plated into 96-well flat-bottomed microtiter plates at a density of 2.5 × 10^4^ cells per well. After 3 to 4 days, confluent monolayers were washed with culture media and incubated with SP/MEC-CM at three different SP/MEC ratios for 48 hrs. Next, the cells were washed and fixed in 1% paraformaldehyde in PBS for 15 min and then blocked with 2% bovine serum albumin overnight at 4°C. The monolayer was incubated with monoclonal mouse antihuman E-selectin and ICAM-1 antibodies (1 mg/ml; R&D Systems) for 2 hrs at 37°C, washed three times with PBST (PBS with 0.05% Tween-20), and once with PBS, and further incubated for 2 hrs with goat anti-mouse IgG conjugated to alkaline phosphatase (Sigma, Inc.). After additional extensive washing with PBST, the substrate, p-nitrophenyl phosphate, was added, and the optical densities were read at 405 nm.

### 2.11. Assay of Neutrophil-Endothelial Cell Adherence

^51^Cr-labeled neutrophils were added to HUVEC monolayers (10 : 1 ratio of labeled neutrophils: HUVECs) which were preincubated with the 10 SP/MEC-CM for 24 hrs. After incubation for 30 min, the supernatants were removed and collected. The cells were washed twice for the removal of nonadherent HUVEC-neutrophils, and the washed-fluid was collected. The adherent HUVEC-neutrophils were lysed with 2N NaOH and the lysate was collected. A gamma counter (WALLAC 1470 WIZARDTM) was used to assess the ^51^Cr activity of the supernatant, washed-fluid, and lysate. The percentage of adherence for the ^51^Cr-labeled neutrophils to HUVEC monolayers was quantitated as follows: Lysate (cpm) × 100/(supernatant (cpm) + Wash (cpm) + Lysate (cpm)).

To confirm the influence of the IL-8, in the SP/MEC-CM, on the changes of adherence between the HUVECs and neutrophils, 100 *μ*g/ml of goat antihuman IL-8 IgG or an identical amount of goat IgG (isotype control) were added to the 10 SP/MEC-CM before adding the 51Cr-labeled neutrophils to the HUVECs.

### 2.12. Statistical Analysis

All data were obtained from four replicate samples in each experiment. The statistical analysis was performed using SAS statistical software. Ordinal variables were compared with a paired t-test and the Kruskal-Wallis test. Data are presented as mean with standard deviation (Mean ± SD). Statistical significance was set at the 5% probability level.

## 3. Results

### 3.1. Induction of IL-8

**Figure 1:**
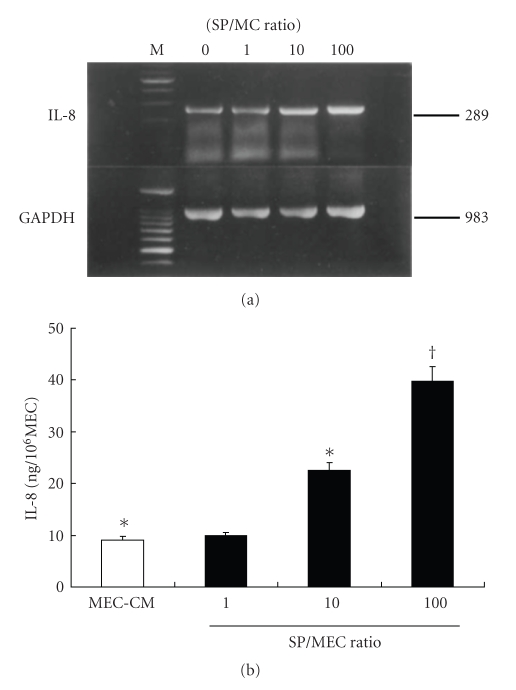
The kinetics of IL-8 with alteration of SP/MEC ratio. expression of IL-8. IL-8 mRNA expression (a) and secreted IL-8 concentration (b) were significantly increased in the 10 and 100 SP/MEC-CM compared with the MEC-CM in an SP number-dependent manner. ^∗,†^ *P* < .01.

IL-8 was detected in the MEC-CM and in all SP/MEC-CM; however, IL-6 and TNF-*α* were not detected in any of these conditions. The expression of IL-8 mRNA and the concentration of IL-8 were significantly increased in the 10 and the 100 SP/MEC-CM compared with MEC-CM in a SP number-dependent manner (*P* < .01) (Figures 1(a) and 1(b)).

### 3.2. Kinetics of IL-8 Production

**Figure 2:**
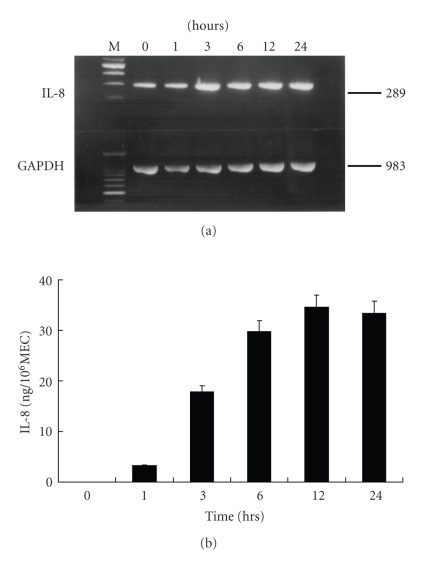
The kinetics of IL-8 with time. The expression of IL-8 mRNA (a) continued to increase from 3 hrs to 24 hrs after incubation. The concentration of secreted IL-8 (b) was significantly increased from 3 hours and peaked at 12 hours after incubation.

To evaluate the kinetics of IL-8 production in the 10 SP/MEC-CM, changes in the expression of IL-8 mRNA and the concentration of IL-8 were determined using RT-PCR and ELISA in length of time. The IL-8 mRNA expression continued to increase from 3 hours until 24 hours after the initiation of incubation and the level of IL-8 also significantly increased from 3 hours and peaked at 12 hours after incubation (Figures 2(a) and 2(b)).

### 3.3. The Expression of CD11b/CD18 (Mac-1) on Neutrophils

**Figure 3:**
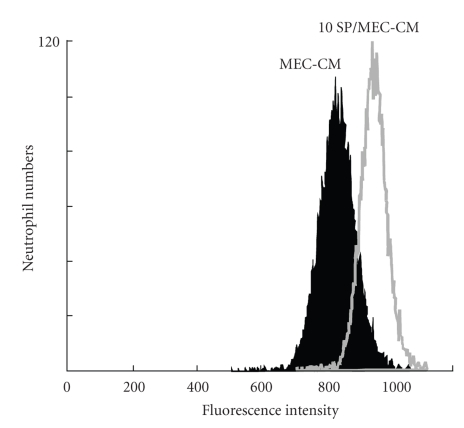
Flow cytometric analysis of the expression of CD11b/CD18 on neutrophils. The expression of CD11b/CD18 on neutrophils was significantly higher in the 10 SP/MEC-CM than in the MEC-CM (*P* < .05).

**Figure 4:**
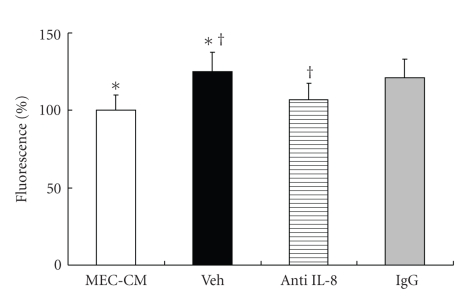
Changes of the expression of CD11/CD18 by anti-IL-8 antibody. The percentage of fluorescent intensity was significantly higher in the 10 SP/MEC-CM (Veh group) than in the MEC-CM. The fluorescent intensity in SP/MEC-CM treated with anti-IL-8 antibody was significantly less than that in Veh group. ^∗,†^ *P* < .05.

The fluorescent intensity for CD11b/CD18 on neutrophils was significantly higher in the 10 SP/MEC-CM than in the MEC-CM (*P* < .05). The peak fluorescent intensity shifted from 760 in the MEC-CM to 890 in the 10 SP/MEC-CM (Figure 3). The percentage of fluorescent intensity in the 10 SP/MEC-CM (Veh group) was significantly higher than in the MEC-CM; the increment was significantly blocked by anti-IL-8 antibody (*P* < .05) (Figure 4).

### 3.4. The Expression of the Endothelial Adhesion Molecules (E-Selectin and ICAM-1)

**Figure 5:**
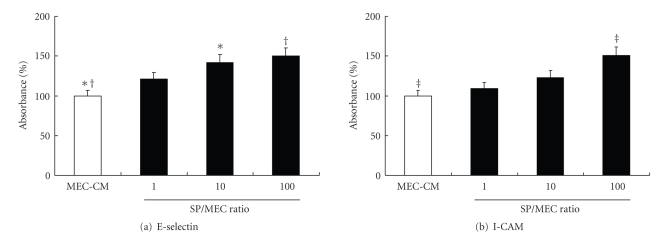
ELIZA for endothelial adhesion molecules (E-selectin and ICAM-1) with alteration of SP/MEC ratio. The endothelial expressions of E-selectin and ICAM-1 were increased in an SP number-dependent manner. The expression of E-selectin was significantly increased in the 10 and 100 SP/MEC-CM and ICAM-1 in the 100 SP/MEC-CM. *P < .05, ^†, ‡^ *P* < .01.

**Figure 6:**
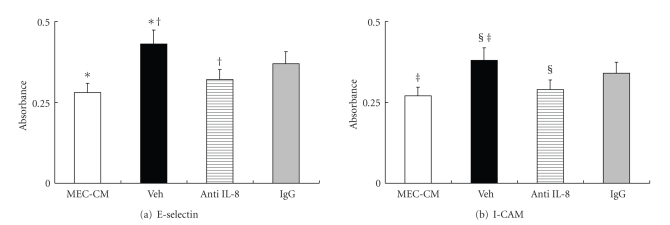
Changes of the expression of endothelial adhesion molecules (E-selectin and ICAM-1) by anti-IL-8 antibody. The expression of E-selectin and ICAM-1 on HUVECs were significantly increased in the 10 SP/MEC-CM (Veh group) compared with the MEC-CM. The expression of E-selectin and ICAM-1 on HUVECs in 10 SP/MEC-CM treated with anti-IL-8 antibody was significantly less than that in Veh group.   ^∗, †, ‡^ *P* < .05, ^§^ *P* < .01.

The absorbance at 405 nm of E-selectin and ICAM-1 on HUVECs was increased in an SP number-dependent manner. The absorbance of E-selectin was significantly increased in 10 and 100 SP/MEC-CM and ICAM-1 in the 100 SP/MEC-CM (Figure 5). The absorbance of E-selectin and ICAM-1 in the 10 SP/MEC-CM (Veh group) was significantly increased compared with the MEC-CM (*P* < .01), and the increment was significantly blocked by anti-IL-8 (*P* < .01 in E-Selectin, *P* < .05 in ICAM-1) (Figure 6).

### 3.5. Adherence between Neutrophils and Endothelial Cells

**Figure 7:**
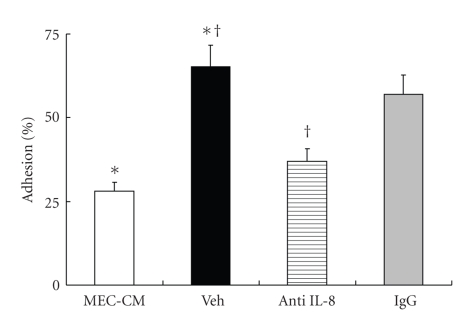
The radiosorbent assay for adherence between neutrophils and HUVECs. The percentage of adherence between radiolabeled neutrophils and endothelial cells was significantly increased in the 10 SP/MEC-CM (Veh group) compared with the MEC-CM. The percentage of adherence between radiolabeled neutrophils and endothelial cells in the 10 SP/MEC-CM was significantly less than that in Veh group.   ^∗, †^ *P* < .01.

The percentage of adherence between radiolabeled neutrophils and HUVECs was significantly increased in the 10 SP/MEC-CM (Veh group) compared with the MEC-CM (*P* < .01) and the increment was significantly blocked by anti-IL-8 antibody (*P* < .01) (Figure 7).

## 4. Discussion

Long-term low-dose macrolide therapy is less effective in patients with high serum IgE or marked eosinophilia in nasal smear, as in allergic CRS. It is more effective in non-allergic CRS patient with persistent purulent discharge [3, 6]. One of the prominent features of this disease is persistent purulent sinus effusion containing numerous neutrophils [2, 3]. It has been known that macrolides block the positive feedback loop of IL-8 and resulting in clinical efficacy in chronic airway inflammation [3, 7, 8].

Recent advances in sinusitis research have revealed two positive feedback mechanisms that explain chronic neutrophil accumulation in the sinuses [3]. One is the IL-1*β* system. The adherence between the circulating neutrophils and vascular endothelium is a critical step in the emigration of neutrophils through blood vessel walls to the inflammatory sinus mucosa. IL-1*β* is related with the expression of vascular adhesion molecules, such as ICAM-1 and E-selectin. The other is the IL-8 system. In the initial phase, the mucosal epithelial cells that recognize the microbes secrete IL-8. The IL-8 in the sinus effusion initiates neutrophil exudates and the emerged neutrophils in the sinus effusion also secrete IL-8. And then the newly secreted IL-8 elicits further migration of neutrophils out of the mucosa into the sinus effusion. Proteases and superoxides released from the recruited neutrophils lead to the impairment of mucociliary function followed by the retention of sinus effusion.

To investigate the changes of inflammatory cytokines from MEC after bacterial infection, we measured the level of cytokines in MEC-CM and SP/MEC-CM at three different SP/MEC (1/1, 10/1, 100/1) rations. IL-6 and TNF-*α* were not detected in any of the conditions, but IL-8 was detected in all of the conditions. That means the IL-8 secreted from the MECs is the key cytokine for development and maintenance of non-allergic CRS.

We made further studies into IL-8 and showed that the expression of IL-8 mRNA and the concentration of secreted IL-8 were increased in an SP number-dependent manner. Moreover, the changes of IL-8 mRNA and secreted IL-8 were in parallel with each other in length of time. These results demonstrated that MECs secrete newly formed IL-8 immediately after bacterial infections. 

Vascular adhesion and transendothelial migration of neutrophils are also important steps in neutrophil recruitment in nonallergic CRS. There have been some reports suggesting that the IL-8 increases the expression of the adhesion molecules on leukocytes. In vitro, it was found that IL-8-induced human polymorphonuclear leukocytes (PMNLs) migration across unstimulated human umbilical vein endothelial cells (HUVECs) which was mediated by the CD18 (beta2) integrins, LFA-1 and Mac-1 [9]. The IL-8 has been also proven to increase the adhesion capacity of monocytes by triggering conformational changes in Mac-1 on the rolling monocytes [10]. Our study showed that the IL-8 increased the Mac-1 expression on neutrophils and enhanced the neutrophils adhesion to the endothelial cells. These results are in agreement with those of previous studies [9, 10].

We demonstrated that the increment of the expression of E-selectin and ICAM-1 in the 10 SP/MEC-CM (Veh group) HUVECs was significantly blocked by anti-IL-8, and then it seemed that the increment was responsible for IL-8. However, there has been no prior report proving that the IL-8 directly enhances the expression of vascular adhesion molecules. Several reports have shown autocrine and paracrine role for IL-8 in modulating endothelial cell proliferation [11, 12]. It also has been reported that neutralizing antibodies to IL-8 inhibit endothelial cell proliferation by increasing apoptosis of endothelial cells [11]. Therefore, we speculated that the increased expression of E-selectin and ICAM might account for the endothelial cell proliferation induced by IL-8 from MECs and HUVECs during bacterial infection and that anti-IL-8 induced apoptosis of the endothelial cells might be responsible for the decrement of E-selectin and ICAM expression. Even though it is obvious that IL-8 is more or less responsible for the expression of E-selectin and ICAM, a limitation of this study is that we cannot rule out the possibility of the presence of other inflammatory cytokines from MECs or HUVECs, such as IL-1*β*. Further molecular studies are needed to define how the IL-8 participates in the expression of vascular adhesion molecules.

## 5. Conclusion

IL-8 is the major factor for neutrophil recruitment in nonallergic CRS. Secreted IL-8 enhanced transendothelial migration of neutrophils by not only increasing the adhesion molecules on neutrophil but also increasing the expression of vascular adhesion molecules. During the initial phase of bacterial infections, IL-8 was derived from the MECs; therefore, we suggested that MECs would be the most important source of IL-8 and recognition of pathogens by sinonasal epithelial cells should be an important initial step for neutrophil recruitment in nonallergic CRS.
